# The Gift of Expression

**Published:** 1920-09-25

**Authors:** 


*To the Editor of* The Hospital.

### The Gift of Expression.

*To the Editor of* The Hospital.

Sir,—In reply to the very clever article, " The Gift of Expression," by a matron, it would appear to be unfortunate that this lady's experience has been apparently among a dull lot of nurses, but, speaking broadly, we cannot expect the rank and file to lead the profession, any more than a general would expect the rank and file of his army to lay down schemes and plans.

Let the leaders of the profession, the College of Nursing and the General Nursing Council, lay down their schemes and give the rank and file an opportunity to criticise them together with the public. How can we expect the rank and file to know what is good for the profession? It is only those who by years of experience based on sound judgment who should attempt to solve the intricate problems with which the profession is faced, and even then it is a case of " where angels fear to tread," etc. At the same time, is it very wise to encourage the members

**The Editor's Letter Box—(*continued*).**

to state their opinions clearly and succinctly as possible by introducing debates, etc.? But by publicly stating their lethargic attitude would not to me appear to be the best method of encouragement, judging from my own experience of nurses, which has been very considerable. I have already stated in one of the nursing papers that *esprit de corps* or the bond of comradeship cannot be imposed on from without—it must be evolved from within; and once you get a hold of the nurses based on grounds of good-fellowship they become very plastic and easily moulded. You will never get this by shouting their lethargic attitude and indifference from the "house-tops," and as a member of the profession I certainly resent this method.—Yours faithfully,

A College Member.

